# Microglial and Astrocytic Function in Physiological and Pathological Conditions: Estrogenic Modulation

**DOI:** 10.3390/ijms21093219

**Published:** 2020-05-02

**Authors:** Andrea Crespo-Castrillo, Maria-Angeles Arevalo

**Affiliations:** 1 Instituto Cajal, Consejo Superior de Investigaciones Científicas (CSIC), Avenida Doctor Arce, 37, 28002 Madrid, Spain; 2 Centro de Investigación Biomédica en Red Fragilidad y Envejecimiento Saludables (CIBERFES), Instituto de Salud Carlos III, 28029 Madrid, Spain

**Keywords:** tibolone, estradiol, neuroinflammation, brain injury, phagocytosis, glial cells, sex differences

## Abstract

There are sexual differences in the onset, prevalence, and outcome of numerous neurological diseases. Thus, in Alzheimer’s disease, multiple sclerosis, and major depression disorder, the incidence in women is higher than in men. In contrast, men are more likely to present other pathologies, such as amyotrophic lateral sclerosis, Parkinson’s disease, and autism spectrum. Although the neurological contribution to these diseases has classically always been studied, the truth is that neurons are not the only cells to be affected, and there are other cells, such as glial cells, that are also involved and could be key to understanding the development of these pathologies. Sexual differences exist not only in pathology but also in physiological processes, which shows how cells are differentially regulated in males and females. One of the reasons these sexual differences may occur could be due to the different action of sex hormones. Many studies have shown an increase in aromatase levels in the brain, which could indicate the main role of estrogens in modulating proinflammatory processes. This review will highlight data about sex differences in glial physiology and how estrogenic compounds, such as estradiol and tibolone, could be used as treatment in neurological diseases due to their anti-inflammatory effects and the ability to modulate glial cell functions.

## 1. Introduction

One of the most sensitive and precise defense systems against trauma, bacteria, and viruses is the immune system, which extends throughout the body. Until a few decades ago, it was thought that the brain was a privileged organ that did not have an immune system [1]. However, this concept has currently changed, and the presence of the blood–brain barrier (BBB), specialized immune cells, and a system that connects the brain to the peripheral circulation, known as “glymphatic system” [2,3,4], make us understand that the brain is able to respond against injuries in an efficient way. The response of specialized cells to brain injury trying to fight damage and then restoring the brain parenchyma is known as neuroinflammation. Neuroinflammation has been widely studied, and it is currently known that neuroinflammation has different faces depending on the time of activation [5,6]. Hence, in an acute stage, neuroinflammation has beneficial effects in recovering homeostasis in the central nervous system (CNS), and it is able to face aggressions such as brain injury, trauma, hypoxia, or bacterial and viral infections. Different cell types participate in the neuroinflammatory response, including glial cells, endothelial cells, and neurons. In addition, BBB is usually damaged, and there is an increase in permeabilization through which peripheral immune system cells can enter into brain parenchyma. The cells that usually penetrate the CNS are monocytes, macrophages, dendritic cells, and T lymphocytes [7,8,9].

Many diseases of the nervous system, such as major depression, Alzheimer’s disease, autism spectrum, Parkinson’s disease, and multiple sclerosis, present an exacerbated inflammation or an incorrect response of the immune system in the CNS, so the severity of the pathology could be related to inflammatory processes [10]. Thus, an incorrect control of neuroinflammation, such as when it is prolonged over time, making it too aggressive and producing too many proinflammatory factors such as interleukin 6 (IL-6), interleukin 1β (IL-1β), or tumor necrosis factor (TNFα), or when it appears for no apparent reason, as in autoimmune diseases, could contribute to the etiology of neurological disease [11].

Curiously, in many of these pathologies, sex differences can be found [12,13,14,15]. Sex differences can be found not only in response to pathological conditions but also under physiological conditions. In physiology, there are basic genetic differences. For instance, the SRY gene on the Y chromosome is responsible for the development of the testes that produce testosterone, which reaches the brain, where it is transformed into estradiol by the aromatase enzyme, with estradiol being responsible for the masculinization of the brain [16,17,18,19,20]. In fact, it has been shown that estradiol levels are different in male and female brains during a developmental period around birth, and there are well-established sex differences in the amount of testosterone and estradiol in the hypothalamus and preoptic area during the perinatal period. Testosterone declines in both sexes with increasing age, but its action on the brain persists during life [21]. One interesting tool to study the genetic/sex hormone contribution to pathologies is the use of the four core genotype (FCG) model [22]. Using this model, researchers were able to discover recently why women are more likely to develop multiple sclerosis [23]. For this reason, it is easy to think that not only do cells respond differently in males and females after an insult but that sexual differences can also be found in cell performance under normal conditions due to their different genotype.

Furthermore, although sexual behavior in males and females is mainly controlled by neuronal circuits [24,25,26,27], some studies have shown how these sex differences could be sculpted by microglia and astrocytes [28,29]. Hence, glial cells could play a key role in modulating sex differences in the brain. In this review, we present a description about several sexual differences that can be found in both physiological and pathological conditions on glial cells and a description about the impact of estrogenic compounds like estradiol and tibolone on astrocyte and microglia functional activity.

## 2. Microglia

Microglia are considered to be the immune cells in the brain, and their origin is mesodermal [30,31,32,33,34,35]. They infiltrate to the CNS during gestation of embryo, and they develop from PU.1 + cells [36,37]. Although the number of microglial cells is small in the embryo state, they are able to proliferate by the induction of colony-stimulating factor 1 (CSF1) and colonize the whole brain [30,38].

Microglial cells play a key role during neural development. One of the functions they have in developmental stages is to perform synaptic pruning, which consists of eliminating defective, immature, or redundant synaptic buttons. It has been shown that the alteration of this function of microglia during development can contribute to the appearance of pathologies in adults, such as autism, depression, or learning difficulties, due to the lack of elimination of inappropriate neuronal connections between the cortex and the hippocampus [39,40].

Microglial morphology is an important characteristic to understand cell function [41]. Although there are many variations, some studies have shown how size and shape correlate with different microglial functions. In a resting tissue state, microglia are present as a “resting” phenotype with a small soma and long ramifications, whose function is to scan the environment around them [42,43]. In pathological conditions, microglia acquire an amoeboid morphology. Along the cell body, including processes, chemokine (C-X3-C motif) receptor 1 (CX3CR1) is widely expressed in microglial cells. This is a chemokine receptor that is only expressed in the brain by microglia, and it is activated by fractalkine (CX3CL1, chemokine (C-X3-C motif) ligand 1) expressed by neurons. Through this receptor, microglia surveil the state of the neural connections, being able to not only act in case of damage but also modulate and maintain dynamic and constant monitoring of all circuits [44,45].

As indicated above, there are sex differences in many neurological diseases. Microglia are altered in all of them, but sexual differences can also be found in healthy state [46]. In Table 1, some of them are indicated with their references. For example, microglial development in males is delayed compared to females [47]. There are also differences in the number of cells in the early postnatal stages in which the males have a higher number of cells, while it is the females that have a higher number of microglial cells in the adult stages [48,49]. Differences in cell morphology and phagocytic capacity are also present and dependent on the brain region. In embryonic stages, there are no morphological or phagocytic differences between male and female microglial cells in amygdala or hippocampus. However, in neonatal stages, males are more prone to present an amoeboid morphology and phagocytic cups than female microglia in amygdala. On the other hand, in the hippocampus, males again present an amoeboid morphology, but female microglia have more phagocytic cups and CD68+ cells than male microglia. In adult stages, male and female microglia are similar [29,50]. In in vitro experiments, many studies have shown that female microglia have a higher phagocytic capacity than males [51,52]. Furthermore, in general, transcriptomes show that males express more genes related to inflammation and females express more genes associated to cell repair [49,53]. All these physiological sex differences have been recently reviewed by Bordt et al., Yanguas-Casas, and Villa et al. [50,54,55].

Microglia could also play a relevant role in the difference in social behavior seen in men and women in adult age. There is a sexual difference in the preoptic area (POA), where microglia density and activation are higher in males than in females due to testosterone increase in the brain. This sex hormone is important to masculinize and maintain male sexual behavior in adulthood by modifying the neuronal circuits and the morphology of the microglia in a dimorphic way [28,98].

## 3. Microgliosis

After brain injury, stroke, or bacteria and virus invasion, microglia change the phenotype to an activated or reactive phenotype, characterized by shorter cytoplasmic processes and a bigger soma size. It is important to understand that the microglial phenotype is not an all-or-nothing result but that there is instead a wide spectrum where, depending on the triggering brain area or cytokine release, different shapes and sizes can be found [99,100,101]. In addition to activated or resting microglia, there is a phenotype called “phagocytic phenotype”, where microglia present a thick cell body and long ramification with phagocytic cups. This phenotype can be found in both healthy and disease conditions [102,103]. Microglia activation is a complex and very well-regulated process (for review, see [42,104]).

When damage occurs in the cerebral parenchyma, microglia will move fast to that point [105], although in some cases the detection of a significant increase of proinflammatory cytokines occurs at 24 h after injury. Using immunochemistry techniques, it has been shown that there is an activation peak between two and seven days after injury [106,107,108,109,110]. It has been determined that adenosine triphosphate (ATP) is capable of attracting microglia [105] and that blocking purinergic receptor with P2X inhibitors makes it possible to inhibit microglial migration. P2X4, P2Y1, and P2Y12 are essential in microglia chemotaxis [111,112], and it has recently been shown that P2Y12 plays a key role by not only being activated after damage but also controlling neuronal mitochondria state to regulate synapsis and neuronal death through a new structure named purinergic junctions [113]. On the other hand, although microglia are able to detect this ATP directly, some studies have shown that, after brain damage, astrocytes are the first cells to detect it, and astrocytes will in turn release more ATP than what will be detected by microglia [105].

Another stimulus that will cause microglia activation and migration is monocyte chemoattractant protein 1 (MCP-1) or chemokine C-C motif ligand 2 (CCL2) produced by neurons. In turn, the microglia will release more MCP-1, which attracts more cells to the damaged area [114]. In addition, other factors contribute to the regulation of migratory stimuli, such as macrophage inflammatory protein 1-alpha (MIP-1α), also known as chemokine C-C motif ligand 3 (CCL3), and RANTES (regulated on activation, normal T cell expressed and secreted), also known as chemokine C-C motif ligand 5 (CCL5). By binding to their receptors, these ligands will facilitate the release of cytokines, such as IL-1β, IL-6, and TNFα [42,104,110,115,116], amplifying the proinflammatory signal.

**Figure 1:**
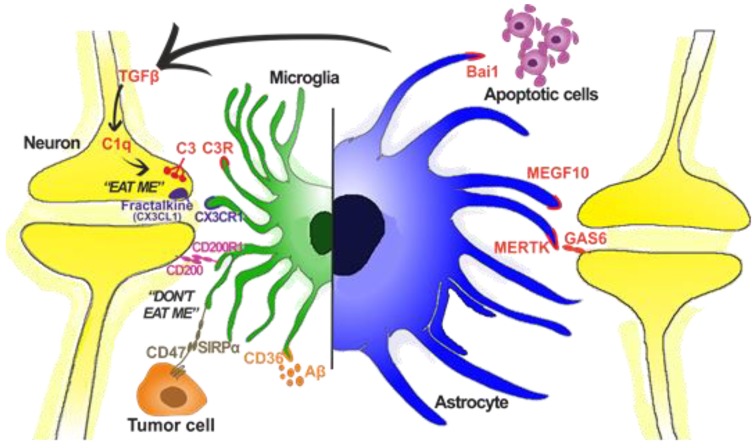
Graphical representation of different phagocytic receptors and ligands in microglia and astrocytes. Although microglia are considered the professional phagocytic cell of the brain, astrocytes are also able to modulate neuronal synapsis. In the case of microglia, there are stimuli for both “eat me” and “don’t eat me” signals. Apart from neurons, they are also able to modulate tumor cells (microglia), apoptotic cells (both), and Aβ intake (microglia and in vitro astrocytes). Based on [122,133,134,135].

Microglia also have specific receptors that control phagocytosis (Figure 1). Purinergic receptor P2Y6 (platelet purinergic receptor 6) will regulate it and will be activated when neuronal damage is produced [117,118]. Fractalkine (CX3CL1), mentioned before, not only activates migration [119,120,121] but is also a prophagocytic stimulus. Damaged neurons will express fractalkine and thus attract microglia to the injured site, which will phagocyte the neuronal debris or the altered neuronal connections, allowing the neuronal circuitry to function properly. Fractalkine can also be expressed by astrocytes in vitro and after damage situations like in experimental autoimmune encephalomyelitis (EAE) and in a neuropathic pain model, even if they do not express their CX3CR1 receptor [122,123,124,125,126,127]. Microglia also have the triggering receptor expressed on myeloid cells 2 (TREM2) in the cell membrane. This receptor is closely related to phagocytosis in microglia, and it has been observed that the phagocytic capacity of microglia is inhibited in knockout (KO) mice of TREM2 [128,129,130] (for review, see [131]). Binding ligands of TREM2 are anionic carbohydrates, lipopolysaccharide (LPS) and apolipoprotein E (ApoE). This receptor is also relevant in Alzheimer’s disease, so much that it has been considered as an early marker of the disease [132].

After brain injury or trauma, sex differences can also be found in reactive microglia (for review, see [136]). Male microglia produce more arginase-1 and more neuroglobin than female microglia after a penetrating brain injury. This will lead to a difference in neuronal death between sexes being reduced in males [71]. However, after a stroke, males express more CD16/32, while females express more anti-inflammatory protein Ym1, having a better outcome of the disease [53]. In in vivo stress models, microglia activation is different depending on the sex [66,76,77]. Therefore, in some in vitro models, male and female microglia have different migratory, phagocytic, and motility capacity in both basal conditions and after interferon (IFN) and LPS treatment [51,64]. In males, microglia mediates neuropathic pain, while microglia are not implicated in females [78,79,80,81].

## 4. Astrocytes

Astrocytes are the most abundant glial cells in the brain [137]. Unlike microglia, whose embryonic origin is mesodermal, astrocytes are ectodermal, like neurons. Astrocytes mostly derive from the radial glia, which is necessary for neuronal migration in embryonic stages. In adult stages, they help in the migration of olfactory bulb neurons [138,139].

In addition, numerous results have shown that astrocytes participate in glutamatergic synaptic function, forming what has been called the “tripartite synapse” [140]. Here, neurons and astrocytes work together to carry out the glutamate–glutamine cycle to precisely control the amount of neurotransmitter that is emitted to the postsynaptic neuron. There has been much debate in this regard as there are studies that suggest that this synapse formation only appears in the developing brain and not in adulthood [141] (for review, see [142]).

Like microglia, astrocytes have a very important role in eliminating synaptic connections by phagocytosis, both in physiological processes and in pathological conditions (Figure 1). The most studied routes by which astrocytes carry out phagocytosis are MERTK (Mer receptor tyrosine kinase) and MEGF10 (multiple epidermal growth factor (EGF)-like-domains 10). Both routes begin with classic signs of cell death recognition, such as phosphatidylserine in the membrane, although they are also able to phagocyte active connections, thus modulating neuronal circuitry [143,144] (for review, see [134]). Astrocytes also have the capacity to engulf apoptotic cells by brain-specific angiogenesis inhibitor 1 (BAI1) receptor [145] (for review see [135,146]).

Furthermore, astrocytes are also important for the maintenance of the BBB, controlling blood flow, and modulating nutrient uptake, such as oxygen and glucose, from the bloodstream according to the needs of the brain at any time [7,9,147,148].

Astrocytes present some sex differences in physiological conditions, and most of them depend on the brain region. In Table 1, these differences are summarized with references. For example, in the hypothalamus, males express a greater number of cells compared with females [84,85,87]. Besides, the cell morphology is different as male astrocytes present more stellated morphology while female astrocytes are more bipolar in in vitro models [87]. These morphological differences could be regulated by Notch signaling, at least after an inflammatory challenge [88]. In the hippocampus, the astrocyte morphology is similar to that of the hypothalamus, but females express more glial fibrillary acidic protein (GFAP) in this region than males, which could indicate a higher cell number [82]. Some studies have shown that testosterone can regulate cell number differences observed in the hypothalamus. Removing testosterone in males or adding it to females reduces and increases GFAP levels, respectively [83]. It has also been shown that male astrocytes have a greater respiratory capacity than female ones at low physiological level of oxygen, which could implicate a different modulation of mitochondria respiratory chain. It could generate some sexual differences observed in some neurodegenerative diseases [89]. Differences in the estrogenic concentration between males and females could lead to these observed differences. It has already been reviewed [149] how estradiol can sculpt functional and behavioral sex differences by modulating astrocytes directly or through microglial cells and their crosstalk with neurons [29,150].

As with microglia, it is wrong to consider astrocytes found in a physiological environment as inactive cells as both astrocytes and microglia are essential cells for the proper functioning of CNS in physiological conditions. Even so, when a pathophysiological event occurs, the function and morphology of astrocytes will change, producing an astrocytic activation or astrogliosis [151,152].

## 5. Astrogliosis

Astrocytes will lead to different types of astrogliosis, which may be more moderate or more severe depending on the level of damage. The main difference between them is that there is overexpression of proinflammatory and GFAP genes in moderate astrogliosis, but there is no proliferation and overlapping between astrocytes. Instead, in severe astrogliosis, where proinflammatory genes are activated and GFAP increases its expression, there is an activation of cell proliferation and cells overlap over other astrocytes, thus eliminating their contact inhibition [151].

In astrogliosis, astrocytes do not act alone but rather always work together with microglia and NG2 cells (neural/glial antigen 2), whose relationship in both health and disease has been recently and extensively reviewed by Vainchtein and Molofsky [153]. In addition, microglia can activate astrocytes, leading them to a proinflammatory state (A1) [154]. Astrocytic activation can occur due to neurodegenerative diseases, viral or bacterial infections, trauma, and brain injuries. In this last case, a glial scar will be formed [151,155].

One of the first consequences of damage in the cerebral parenchyma is the rupture of the neuronal connections, which will produce demyelination and axonal degeneration [156,157,158]. Later, in a second phase, there will be a first wave of neuronal death that will coincide in time with the vascular cascade (increased flow and opening of BBB) and the release of molecules, which will activate glia (astrocytes, microglia and oligodendrocytes), pericytes, and fibroblasts. In this second phase after an injury, the glial cells will release cytokines and chemokines, which will attract cells from outside the CNS, especially from the peripheral immune system. These cellular responses after injury are necessary to face damage, but if they are not carried out in a controlled manner and the reaction lasts too long, the glial cells themselves can contribute to increased neuronal damage, which makes the damage worse [158,159].

As indicated above, when an injury occurs, microglial cells, astrocytes, and also the NG2 glia will proliferate and migrate to the damaged area. Astrocytes are able to proliferate, but they need numerous stimuli, such as EGF, fibroblast growth factor (FGF), endothelin-1, and ATP [155,160]. Classically, it has been considered that this proliferation in the injured area will lead to what is known as a glial scar. However, despite the name, several studies have shown that the percentage of glial cells, especially in the core of this scar, is really low in comparison to the amount of cells from the extracellular matrix (such as pericytes, fibroblasts, and ependymal cells), which increases the concentration of fibronectin, laminin, and collagen [161].

On the other hand, many astrocytes are observed in the periphery of the lesion (penumbra area). It has long been considered that the glial scar will inhibit neuronal regeneration, but more and more studies suggest that this scar helps regeneration. The scar increases the availability of TGF-β, which not only increases the release of neurocam and inhibits neuronal growth but also produces the deactivation of the microglia, turning it to a more restorative phenotype that will eventually cause an increase in neuronal regeneration [162,163,164,165]. In addition, it has been shown that, in STAT3 KO animals, which do not have the capacity to form the glial scar, neuronal loss increases and hinders brain recovery [166]. The increased number of glial cells surrounding the fibrotic scar produces many neurotrophic factors, and they also eliminate cellular debris and repair the BBB [146,158,167,168,169].

After physiopathological events, astrocytes also present some sex differences (Table 1). After ischemic injury, there is a sex dimorphism in the astrocytic reactivity and number. Besides, females present an increased amount of Ca^2+^ release [94,95,96]. Upon brain injury, female astrocytes have lower levels of MCP-1 (CCL2), so there is a reduction in the peripheral recruitment [71]. Besides, astrocytes present a sex-specific response after LPS treatment, with male astrocytes having higher proinflammatory gene expression, such as IL-6, TNFα, and IL-1β, than female astrocytes [97]. In addition, male and female astrocytes have a dimorphic response to fatty acids intake, with males presenting a higher proinflammatory profile compared to females [93]. Regarding astrocytic phagocytosis, there have not been too many studies about how sex could modulate it. Nevertheless, we recently described that there was no basal sexual differences in an in vitro model of astrocytic phagocytosis, although they had different responses after a proinflammatory stimulus [90].

## 6. Estradiol and Estrogen Receptor Signaling

As described in the introduction and the following paragraphs, sex hormones could explain the maintenance of sex-specific features seen in microglia and astrocytes. However, it is important to know that, although the “hormone theory” is the most established theory, there are also other factors that could produce these sexual differences. Genetic influence is considered as an extrinsic factor in sex differentiation because some differences appear before hormone production [170]. Besides, some studies have shown that environmental factors could play an important role in influencing microglia in a sex-dependent manner, and this could be important in the onset of many brain diseases. For example, maternal microbiome is able to change microglia transcriptome in fetal stages [61], and air pollution exposure in prenatal age is able to affect microglia, especially in males, providing a possible clue about why males are more prone to bearing autism spectrum disorder in comparison to females [62].

However, setting this aside, many laboratories have demonstrated that it is undeniable that sex hormones drive most of the sex differences observed in the brain. Therefore, it is important to continue studying the relationship between sex steroid hormone production and its influence on glial cells. There are three types of estrogens: estriol and estrone from androstenedione and estradiol from testosterone [171]. All of them present a neuroprotective effect [172,173,174]. Among all estrogens, it is necessary to highlight estradiol because it is one of the hormones whose effects have been studied most in the brain in both development and aging, and its concentration in the brain is higher than estrone and estriol [20,174,175,176].

The main function of estradiol is not to produce sex differences per se. Surely, these differences are a consequence produced by many factors. Sex differences sometimes converge in the same endpoint in males and females, although they are carried out through different mechanisms. However, the endpoint is sometimes is different in males and females, creating the observed differences, for example, in stress response, food intake, odor detection, etc. [177]. Estradiol is produced along the lifespan in most tissues in the mammal body and, along with other sex hormones like progesterone, it is in charge of maintaining the estrous cycle in females and sexual behavior [24,178].

Estradiol is a sex hormone produced mostly in ovaries, although it is also synthesized in less concentration in the bone, adipose tissue, and the CNS of both males and females. Therefore, the brain is also considered a steroidogenic organ as it has all the enzymes necessary to produce estrogens and other steroids locally [179]. To do this, cholesterol is transported to the mitochondria, where it is converted to pregnenolone, which is translocated into the endoplasmic reticulum. Here, after several enzymatic conversions, estradiol is produced from testosterone by aromatase enzyme [180].

The role of aromatase in cognitive function is increasingly important because of studies linking the decreased levels of estradiol in women treated with enzyme inhibitors or after menopause with increased risk of some neurodegenerative diseases like Alzheimer’s disease [181]. In the specific case of brain trauma or injury, it has been reported that there is an increase in the expression of the aromatase enzyme in the damaged areas of the CNS and consequently an increase in estradiol levels, which contributes to fighting brain damage and activating processes that help to repair brain parenchyma. Regarding the cell type, only neurons express aromatase in physiological conditions; however, enzyme expression is induced in astrocytes, but not in microglial cells, by different forms of brain injury [182].

**Figure 2:**
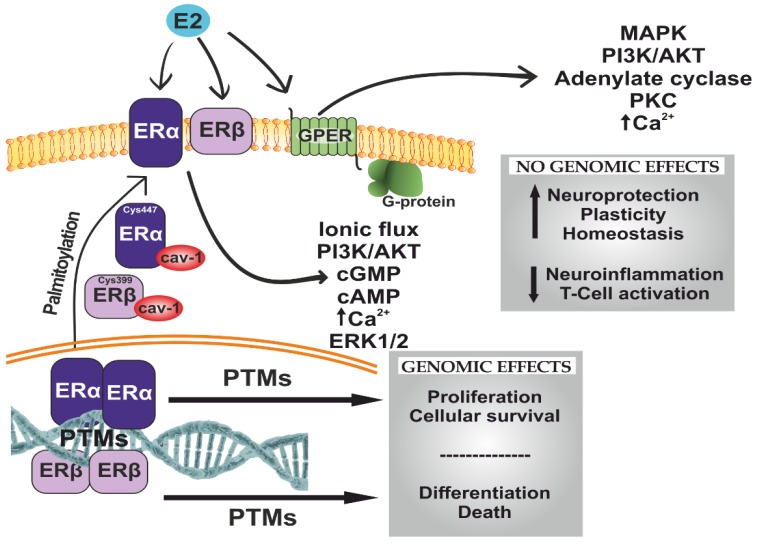
Schematic representation of the effects of estrogen receptors (ERs) after estradiol binding. ERα and ERβ need to dimerize to bind to DNA estrogen response elements after activation with estradiol. These dimers could be homodimers or heterodimers, but the proportion of homodimers is higher. The effects of ERs depend on the tissue they are in, the proportion of each type of receptor, and the crosstalk between them. In general, ERβ antagonize ERα, with different effects on tissue. Furthermore, there are some post-translational modifications (PTMs), both before receptors attach to DNA and after that. Palmitoylation in Cys447 in ERα and Cys399 in ERβ allows them to interact with caveolin-1 (cav-1) and be translocated to the membrane as monomers. G protein-coupled estrogen receptor (GPER) is a receptor associated to Gαq proteins, triggering numerous nongenomic effects. Based on [191,192].

Estradiol exerts its effects by activating different estrogen receptors (ERs) (Figure 2). Both microglia and astrocytes express the necessary receptors to their activation by estradiol. Classically, two types of receptors have been identified: estrogen receptor alpha (ERα) and estrogen receptor beta (ERβ). These receptors are transcription factors that are activated when the ligand (estradiol) binds to them. Once activated, they recruit activating cofactors or transcription repressors and bind to estrogen response elements in the DNA promoter region of certain genes [183]. ERα and ERβ receptors may also be transiently associated with the plasma membrane where, once estradiol binds to them, they regulate several signaling pathways, such as those of PI3K/Akt, ERK, or Jak/STAT. In addition, there are estrogen receptors that are specifically associated with the plasma membrane or endoplasmic reticulum, best known as GPR30/GPER1, which is a receptor associated to Gαq proteins and activates the ERK and PI3K pathways [184,185,186,187,188]. Depending on the tissue where these receptors are, there will be a different response after their activation. In the brain, estrogen receptors are located in neurons and glia cells, and they exert neuroprotective effects. In studies performed in rats, there were no sexual differences in the global expression of estrogen receptors in the brain [189], although in a study on the brain of lambs, ERα had higher expression in males [190]. On the other hand, estradiol concentration in the hippocampus and cortex presented sexual differences in development and in early postnatal stages in rats [21]. In cells, there have not been many studies measuring the number of ERs in a sexually dependent manner, although there are some sexual differences in ER activation, which we will describe below.

### 6.1. Estradiol Effects on Astrocytes

**Figure 3:**
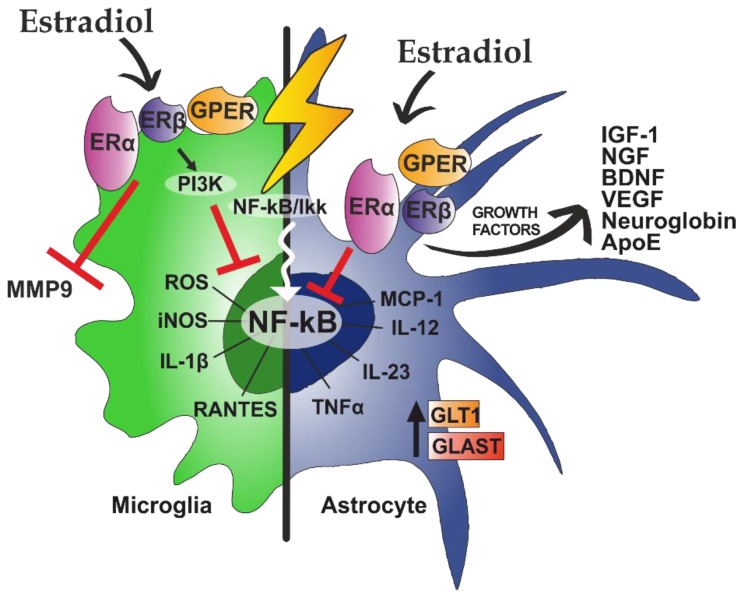
Estradiol has specific actions on microglia and astrocytes. After a proinflammatory stimulus, nuclear factor kappa-light-chain-enhancer of activated B cells (NF-κB) is translocated to the nucleus to activate genes of different proinflammatory pathways. This spreads the damage in the brain by increasing proinflammatory mediators. In microglia, estradiol inhibits the release of metalloproteinase 9 (MMP9). In addition, by activating PI3K, NF-κB translocation to the nucleus is inhibited. In astrocytes, estradiol increases the production of growth factors and glutamate transporters (GLT1 and GLAST) and blocks NF-kB translocation. Based on [196,197,198,199].

Estradiol acts directly on astrocytes through the ERα, ERβ, and GPR30/GPER1 receptors (Figure 3) [193,194,195].

Depending on the experimental model and the type of pathology, estradiol exerts anti-inflammatory actions in astrocytes through different estrogen receptors. For instance, in an experimental multiple sclerosis model, estradiol exerted neuroprotective and anti-inflammatory effects through the ERα receptor. On the other hand, in in vitro models, it acts mainly through ERβ [200]. The anti-inflammatory action of estradiol on astrocytes is mainly mediated by the regulation of nuclear factor kappa-light-chain-enhancer of activated B cells (NF-κB). Nevertheless, estradiol not only blocks p65 nuclear translocation but is also able to repress the NF-κB-dependent transcription of cytokines like CCL2 by activating ERα in astrocytes [201]. In a H_2_O_2_-induced toxicity model, estradiol reduced the secretion of inflammatory mediators in astrocytes [202]. Similarly, estradiol also exerts anti-inflammatory actions by increasing the release of growth factors, such as IGF-1, and decreasing the release of Ca^2+^ [203]. Estradiol also increases the expression of GLT1 glutamate transporters in astrocytes through GPR30/GPER1 receptors, increasing glutamate recapture of the synaptic cleft and thus inhibiting excitotoxicity [204]. In some studies, estradiol treatment was also able to generate sexual differences in astrocytes. In an astrocytic in vitro model, estradiol induced higher increase of intracellular [Ca^2+^] in males than in females and was able to increase progesterone synthesis in male, but not female, astrocytes. Furthermore, estradiol treatment increased the expression of ERα in both sexes but only enhanced the insertion of the receptor into the membrane in females [150]. In astrocyte cultures stimulated with palmitic acid, estradiol had a protective action, increasing the expression of IL-10 in male and female astrocytes. However, estradiol only reduced pJNK, caspase-3, and TNFα levels and apoptotic cell death in male astrocytes [205].

### 6.2. Effects of Estradiol on Microglia

It is also known that estradiol is able to regulate immune response in the brain and has anti-inflammatory properties [206]). Estradiol will bind to ERα receptor, ERβ receptor, and GPER1/GPR30 on microglia (Figure 3) [207,208].

As in astrocytes, estradiol binds to the ERα of microglia and inhibits the transcription factor NF-κB through the activation of PI3K [209,210]. This inhibition will lead to a decrease in the production of nitric oxide synthases (iNOS), which will subsequently reduce the production of nitric oxide (NO) and reactive oxygen species (ROS). Both NO and ROS are responsible for expanding the inflammatory response when damage occurs, so its inhibition has an anti-inflammatory effect [211]. Estradiol will also bind to ERβ and GPER1/GPR30 microglial receptors, thus modulating the release of inflammatory mediators and reducing microglial activation [212,213].

In addition to the anti-inflammatory aspect of estradiol on microglia, it has also been described that the hormone is capable of modulating the phagocytic capacity of microglia during development [214] and in animal models of Alzheimer’s disease [215].

Although the inflammatory process is beneficial for tissue repair, it is sometimes necessary to modulate it. For this reason, there is a large field of study on compounds that are capable of controlling this inflammation and are beneficial to CNS. Estradiol is one of the hormones whose effects have been most studied in the brain in both development and aging [20,176,216]. Thus, it has been widely observed that estradiol exerts protective actions in animal models of Alzheimer’s disease and Parkinson’s disease and increases the number of living neurons after an ischemic episode [217,218,219].

Estradiol treatment also produces some sexual differences in microglia. In hippocampal neonatal microglia, LPS treatment induced a greater in increase in IL-1β mRNA in males than in females. However, after estradiol treatment, anti-inflammatory effects were produced in male microglia, while a proinflammatory effect was produced in females. Nevertheless, in adult hippocampal microglia, estradiol produced an opposite effect, protecting female microglia but not male microglia [64]. Besides, estradiol treatment was able to affect microglia phagocytosis, reducing it in females to reach the male level [52].

## 7. Therapeutic Potential of Estrogenic Compounds

As mentioned above, estradiol has a neuroprotective effect in many neurological diseases, but the use of this compound in a clinical setting is limited due to its side effects [215,220]. It is also important to consider the age of the animals because some studies have shown that estradiol treatment could have a detrimental effect in elderly animals with a reduced estradiol serum concentration [221,222,223]. Estradiol increases the risk of having breast cancer, ovarian cancer, and endometriosis [224]. Hence, it would be necessary to have compounds with estrogenic beneficial effects but without the side effects. Some of these compounds are selective estrogen receptor modulators (SERMs). Instead of being full agonists or antagonists of estrogen receptors, SERMs are competitive partial agonist/antagonist [225]. Many studies have shown that SERMs have neuroprotective effects and regulate gliosis and neuroinflammation [226,227,228,229,230,231]. Two of the most known compounds are raloxifen and tamoxifen, which are typically used for breast cancer treatment and postmenopausal osteoporosis [232,233]. Some studies have shown that these compounds also have beneficial effects in the brain after peripheral administration as they can cross BBB [234,235,236,237].

In the 1980s, another compound called tibolone started to be used in clinical settings for postmenopausal women. Tibolone is a synthetic steroid used to reduce climacteric symptoms, cognitive deficits, and osteoporosis in women [238,239,240]. After treatment, it takes 30 min to have detectable levels of compound in serum, reaching a peak in 60–90 min. It has a half-life of approximately 7 h [241]. Tibolone is metabolized in the liver, producing three different metabolites. The first two metabolites are 3α- and 3β-hydroxytribolone, which are produced by the actions of enzymes 3α-hydroxysteroid dehydrogenase (3α-HSD) and 3β-hydroxysteroid dehydrogenase (3β-HSD), respectively. They activate ERα and ERβ and are able to easily cross BBB, thus increasing their concentration in the brain [242]. The third metabolite is Δ4-isomer, which is formed by the enzyme 3β-hydroxysteroid dehydrogenase-isomerase. Together with tibolone, this metabolite activates progesterone receptor (PR) and androgen receptor (AR) [240,242,243]. Furthermore, the tibolone is capable of controlling estradiol levels in the tissue by inactivating sulfatase enzymes. This regulation will lead to a reduction of undesirable estrogenic effects, reducing the level of estradiol in the breasts and the endometrium but increasing them in the bones and the brain. Hence, tibolone is classified as a selective tissue estrogenic activity regulator (STEAR) [238,239,240].

Although tibolone is widely used in clinical settings, there have not been too many studies about how this steroid could modulate gliosis and neuroinflammation. In an in vivo model of brain injury, we demonstrated that tibolone reduced the number of astrocytes and microglia seven days post-injury, leading to a reduction of neuronal death 14 days post-injury [244]. Studies on glial cells in vitro have revealed that tibolone reduces oxidative damage [245] and exerts anti-inflammatory actions due to the reduction in the activation of NF-κB [246,247] in both microglia and astrocytes. This will produce an improvement in the cellular survival, which could have a neuroprotective effect in neurons. Recently, we also showed that tibolone was able to increase and modulate astrocytic phagocytosis in both physiological conditions and after LPS administration [90].

## 8. Conclusions and Perspectives

In recent years, new neuroglia mechanisms have been elucidated, and it is clear that neurological diseases are a compendium of numerous factors and cells, including microglia and astrocytes. The neuroprotective action of estrogenic compounds is well described, although their effect on glial cells is already being clarified. In addition, it is important to highlight the therapeutic potential of SERMs and tibolone, which could be used in clinical settings to treat pathologies involving neuroinflammation. In addition to using estradiol as a treatment, it would be interesting to study how endogenous sex hormones are able to modulate neuroglia function. The estrogenic differences in the concentration, receptor distribution, and changes in serum level through the lifespan could be relevant in sex differences observed in many neurodegenerative diseases. Nevertheless, further research is needed to elucidate the specific mechanisms of estrogens and the contribution of microglia and astrocytes to overall progression in neuroinflammatory processes.

## Figures and Tables

**Table 1:** Sex differences in microglia and astrocytes in health and pathology with references.

**Microglia**	**Sex Differences**	**References**
**Health**	Development maturation	[47]
	Cell number and density	[48,49,50,56,57]
	Regional distribution	[58]
	Phagocytic capacity	[29,49,51,52,59,60]
	Transcriptome	[49,53]
	Maternal microbiome influence	[61]
	Air pollution exposure	[62]
**Pathology**	Inflammatory sensitivity and reactivity	[47]
	Cellular repair	[53]
	LPS stimulation	[61,63,64,65]
	Stroke and brain injury	[53,66,67,68,69,70,71,72]
	ApoE expression	[73,74,75]
	Response to stress	[66,76,77]
	Neuropathic pain	[78,79,80,81]
**Astrocytes**	**Sex Differences**	**References**
**Health**	Cell number and density	[56,82,83,84,85,86]
	Cell morphology	[84,87,88]
	Respiratory capacity	[89]
	Phagocytic capacity	[90]
**Pathology**	Response/uptake of glutamate	[91,92]
	Response to fatty acids	[93]
	Stroke and brain injury	[71,94,95,96,97]

## Abbreviations

BBB	blood–brain barrier
CNS	central nervous system
EAE	experimental autoimmune encephalomyelitis
IL-1β	interleukin 1β
IL-6	interleukin 6
TNFα	tumor necrosis factor
CX3CL1	chemokine (C-X3- C motif) ligand 1or fractalkine
CX3CR1	chemokine (C-X3-C motif) receptor 1
MCP-1	monocyte chemoattractant protein 1
CCL2	chemokine (C-C motif) ligand 2
MIP-1α	macrophage inflammatory protein 1-alpha
CCL3	chemokine (C-C motif) ligand 3
RANTES	regulated on activation, normal T cell expressed and secreted
CCL5	chemokine (C-C motif) ligand 5
TREM2	triggering receptor expressed on myeloid cells 2
LPS	lipopolysaccharide
ApoE	apolipoprotein E
MERTK	Mer receptor tyrosine kinase
MEGF10	multiple EGF-like-domains 10
BAI1	brain-specific angiogenesis inhibitor 1
GFAP	glial fibrillary acidic protein
EGF	epidermal growth factor
FGF	fibroblast growth factor
TGF-β	transforming growth factor β
ER	estrogen receptors
PR	progesterone receptor
AR	androgen receptor
PTMs	post-translational modifications
NF-κB	nuclear factor kappa-light-chain-enhancer of activated B cells
MMP9	metalloproteinase 9
PGE2	prostaglandin E2
iNOS	nitric oxide synthases
SERMs	selective estrogen receptor modulators
STEAR	selective tissue estrogenic activity regulator

## References

[1] Medawar P.B. (1948). Immunity to homologous grafted skin; the fate of skin homografts transplanted to the brain, to subcutaneous tissue, and to the anterior chamber of the eye. Br. J. Exp. Pathol..

[2] Iliff J.J., Wang M., Liao Y., Plogg B.A., Peng W., Gundersen G.A., Benveniste H., Vates G.E., Deane R., Goldman S.A. (2012). A paravascular pathway facilitates CSF flow through the brain parenchyma and the clearance of interstitial solutes, including amyloid beta. Sci. Transl. Med..

[3] Yang L., Kress B.T., Weber H.J., Thiyagarajan M., Wang B., Deane R., Benveniste H., Iliff J.J., Nedergaard M. (2013). Evaluating glymphatic pathway function utilizing clinically relevant intrathecal infusion of CSF tracer. J. Transl. Med..

[4] Louveau A., Harris T.H., Kipnis J. (2015). Revisiting the Mechanisms of CNS Immune Privilege. Trends Immunol..

[5] Kielian T. (2014). Neuroinflammation: Good, bad, or indifferent?. J. Neurochem..

[6] Ortiz-Rodriguez A., Acaz-Fonseca E., Boya P., Arevalo M.A., Garcia-Segura L.M. (2019). Lipotoxic effects of palmitic acid on astrocytes are associated with autophagy impairment. Mol. Neurobiol..

[7] Zhang C., Brandon N.R., Koper K., Tang P., Xu Y., Dou H. (2018). Invasion of Peripheral Immune Cells into Brain Parenchyma after Cardiac Arrest and Resuscitation. Aging Dis..

[8] Karve I.P., Taylor J.M., Crack P.J. (2016). The contribution of astrocytes and microglia to traumatic brain injury. Br. J. Pharmacol..

[9] Price L., Wilson C., Grant G., Laskowitz D., Grant G. (2016). Blood-Brain Barrier Pathophysiology following Traumatic Brain Injury. Translational Research in Traumatic Brain Injury.

[10] Hong H., Kim B.S., Im H.I. (2016). Pathophysiological Role of Neuroinflammation in Neurodegenerative Diseases and Psychiatric Disorders. Int. Neurourol. J..

[11] Lyman M., Lloyd D.G., Ji X., Vizcaychipi M.P., Ma D. (2014). Neuroinflammation: The role and consequences. Neurosci. Res..

[12] Clayton J.A. (2016). Sex influences in neurological disorders: Case studies and perspectives. Dialogues Clin. Neurosci..

[13] Kim S., Kim M.J., Kim S., Kang H.S., Lim S.W., Myung W., Lee Y., Hong C.H., Choi S.H., Na D.L. (2015). Gender differences in risk factors for transition from mild cognitive impairment to Alzheimer’s disease: A CREDOS study. Compr. Psychiatry.

[14] Werling D.M., Geschwind D.H. (2013). Sex differences in autism spectrum disorders. Curr. Opin. Neurol..

[15] Attarian H., Brandes J., Dafer R., Gerard E., Giesser B. (2015). Sex Differences in the Study of Neurological Illnesses. Behav. Neurol..

[16] Arnold A.P. (2017). A general theory of sexual differentiation. J. Neurosci. Res..

[17] Goodfellow P.N., Lovell-Badge R. (1993). SRY and sex determination in mammals. Annu. Rev. Genet..

[18] Phoenix C.H., Goy R.W., Gerall A.A., Young W.C. (1959). Organizing action of prenatally administered testosterone propionate on the tissues mediating mating behavior in the female guinea pig. Endocrinology.

[19] Jost A. (1970). Hormonal factors in the sex differentiation of the mammalian foetus. Philos. Trans. R Soc. Lond. B Biol. Sci..

[20] McCarthy M.M. (2008). Estradiol and the developing brain. Physiol. Rev..

[21] Konkle A.T., McCarthy M.M. (2011). Developmental time course of estradiol, testosterone, and dihydrotestosterone levels in discrete regions of male and female rat brain. Endocrinology.

[22] Arnold A.P., Chen X. (2009). What does the “four core genotypes” mouse model tell us about sex differences in the brain and other tissues?. Front. Neuroendocrinol..

[23] Voskuhl R.R., Sawalha A.H., Itoh Y. (2018). Sex chromosome contributions to sex differences in multiple sclerosis susceptibility and progression. Mult. Scler..

[24] Micevych P.E., Meisel R.L. (2017). Integrating Neural Circuits Controlling Female Sexual Behavior. Front. Syst. Neurosci..

[25] Rosen R.C., Sachs B.D. (2000). Central mechanisms in the control of penile erection: Current theory and research. Neurosci. Biobehav. Rev..

[26] Coolen L.M. (2005). Neural control of ejaculation. J. Comp. Neurol..

[27] Breedlove S.M. (1985). Hormonal control of the anatomical specificity of motoneuron-to-muscle innervation in rats. Science.

[28] Lenz K.M., Nugent B.M., Haliyur R., McCarthy M.M. (2013). Microglia are essential to masculinization of brain and behavior. J. Neurosci..

[29] VanRyzin J.W., Marquardt A.E., Argue K.J., Vecchiarelli H.A., Ashton S.E., Arambula S.E., Hill M.N., McCarthy M.M. (2019). Microglial Phagocytosis of Newborn Cells Is Induced by Endocannabinoids and Sculpts Sex Differences in Juvenile Rat Social Play. Neuron.

[30] Ginhoux F., Greter M., Leboeuf M., Nandi S., See P., Gokhan S., Mehler M.F., Conway S.J., Ng L.G., Stanley E.R. (2010). Fate mapping analysis reveals that adult microglia derive from primitive macrophages. Science.

[31] Menassa D.A., Gomez-Nicola D. (2018). Microglial Dynamics During Human Brain Development. Front. Immunol..

[32] Hoeffel G., Ginhoux F. (2015). Ontogeny of Tissue-Resident Macrophages. Front. Immunol.

[33] Ginhoux F., Prinz M. (2015). Origin of microglia: Current concepts and past controversies. Cold Spring Harb. Perspect. Biol..

[34] Katsumoto A., Lu H., Miranda A.S., Ransohoff R.M. (2014). Ontogeny and functions of central nervous system macrophages. J. Immunol..

[35] Alliot F., Godin I., Pessac B. (1999). Microglia derive from progenitors, originating from the yolk sac, and which proliferate in the brain. Brain Res. Dev. Brain Res..

[36] Herbomel P., Thisse B., Thisse C. (1999). Ontogeny and behaviour of early macrophages in the zebrafish embryo. Development.

[37] Beers D.R., Henkel J.S., Xiao Q., Zhao W., Wang J., Yen A.A., Siklos L., McKercher S.R., Appel S.H. (2006). Wild-type microglia extend survival in PU.1 knockout mice with familial amyotrophic lateral sclerosis. Proc. Natl. Acad. Sci. USA.

[38] Villa A., Vegeto E., Poletti A., Maggi A. (2016). Estrogens, Neuroinflammation, and Neurodegeneration. Endocr. Rev..

[39] Paolicelli R.C., Bolasco G., Pagani F., Maggi L., Scianni M., Panzanelli P., Giustetto M., Ferreira T.A., Guiducci E., Dumas L. (2011). Synaptic pruning by microglia is necessary for normal brain development. Science.

[40] Neniskyte U., Gross C.T. (2017). Errant gardeners: Glial-cell-dependent synaptic pruning and neurodevelopmental disorders. Nat. Rev. Neurosci..

[41] Davis E.J., Foster T.D., Thomas W.E. (1994). Cellular forms and functions of brain microglia. Brain Res. Bull..

[42] Hanisch U.K., Kettenmann H. (2007). Microglia: Active sensor and versatile effector cells in the normal and pathologic brain. Nat. Neurosci..

[43] Nimmerjahn A., Kirchhoff F., Helmchen F. (2005). Resting microglial cells are highly dynamic surveillants of brain parenchyma in vivo. Science.

[44] Paolicelli R.C., Bisht K., Tremblay M.E. (2014). Fractalkine regulation of microglial physiology and consequences on the brain and behavior. Front. Cell. Neurosci..

[45] Hoshiko M., Arnoux I., Avignone E., Yamamoto N., Audinat E. (2012). Deficiency of the microglial receptor CX3CR1 impairs postnatal functional development of thalamocortical synapses in the barrel cortex. J. Neurosci..

[46] Lenz K.M., McCarthy M.M. (2015). A starring role for microglia in brain sex differences. Neuroscientist.

[47] Hanamsagar R., Alter M.D., Block C.S., Sullivan H., Bolton J.L., Bilbo S.D. (2017). Generation of a microglial developmental index in mice and in humans reveals a sex difference in maturation and immune reactivity. Glia.

[48] Schwarz J.M., Sholar P.W., Bilbo S.D. (2012). Sex differences in microglial colonization of the developing rat brain. J. Neurochem..

[49] Guneykaya D., Ivanov A., Hernandez D.P., Haage V., Wojtas B., Meyer N., Maricos M., Jordan P., Buonfiglioli A., Gielniewski B. (2018). Transcriptional and Translational Differences of Microglia from Male and Female Brains. Cell Rep..

[50] Bordt E.A., Ceasrine A.M., Bilbo S.D. (2020). Microglia and sexual differentiation of the developing brain: A focus on ontogeny and intrinsic factors. Glia.

[51] Yanguas-Casas N., Crespo-Castrillo A., de Ceballos M.L., Chowen J.A., Azcoitia I., Arevalo M.A., Garcia-Segura L.M. (2018). Sex differences in the phagocytic and migratory activity of microglia and their impairment by palmitic acid. Glia.

[52] Nelson L.H., Warden S., Lenz K.M. (2017). Sex differences in microglial phagocytosis in the neonatal hippocampus. Brain Behav. Immun..

[53] Villa A., Gelosa P., Castiglioni L., Cimino M., Rizzi N., Pepe G., Lolli F., Marcello E., Sironi L., Vegeto E. (2018). Sex-Specific Features of Microglia from Adult Mice. Cell Rep..

[54] Yanguas-Casás N. (2020). Physiological sex differences in microglia and their relevance in neurological disorders. Neuroimmunol. Neuroinflamm..

[55] Villa A., Della Torre S., Maggi A. (2019). Sexual differentiation of microglia. Front. Neuroendocrinol..

[56] Mouton P.R., Long J.M., Lei D.L., Howard V., Jucker M., Calhoun M.E., Ingram D.K. (2002). Age and gender effects on microglia and astrocyte numbers in brains of mice. Brain Res..

[57] Rebuli M.E., Gibson P., Rhodes C.L., Cushing B.S., Patisaul H.B. (2016). Sex differences in microglial colonization and vulnerabilities to endocrine disruption in the social brain. Gen. Comp. Endocrinol..

[58] Lawson L.J., Perry V.H., Dri P., Gordon S. (1990). Heterogeneity in the distribution and morphology of microglia in the normal adult mouse brain. Neuroscience.

[59] Weinhard L., Neniskyte U., Vadisiute A., di Bartolomei G., Aygun N., Riviere L., Zonfrillo F., Dymecki S., Gross C. (2018). Sexual dimorphism of microglia and synapses during mouse postnatal development. Dev. Neurobiol..

[60] Wu L.J., Vadakkan K.I., Zhuo M. (2007). ATP-induced chemotaxis of microglial processes requires P2Y receptor-activated initiation of outward potassium currents. Glia.

[61] Thion M.S., Low D., Silvin A., Chen J., Grisel P., Schulte-Schrepping J., Blecher R., Ulas T., Squarzoni P., Hoeffel G. (2018). Microbiome Influences Prenatal and Adult Microglia in a Sex-Specific Manner. Cell.

[62] Bolton J.L., Marinero S., Hassanzadeh T., Natesan D., Le D., Belliveau C., Mason S.N., Auten R.L., Bilbo S.D. (2017). Gestational Exposure to Air Pollution Alters Cortical Volume, Microglial Morphology, and Microglia-Neuron Interactions in a Sex-Specific Manner. Front. Synaptic Neurosci..

[63] Hanamsagar R., Bilbo S.D. (2017). Environment matters: Microglia function and dysfunction in a changing world. Curr. Opin. Neurobiol..

[64] Loram L.C., Sholar P.W., Taylor F.R., Wiesler J.L., Babb J.A., Strand K.A., Berkelhammer D., Day H.E., Maier S.F., Watkins L.R. (2012). Sex and estradiol influence glial pro-inflammatory responses to lipopolysaccharide in rats. Psychoneuroendocrinology.

[65] Doyle H.H., Eidson L.N., Sinkiewicz D.M., Murphy A.Z. (2017). Sex Differences in Microglia Activity within the Periaqueductal Gray of the Rat: A Potential Mechanism Driving the Dimorphic Effects of Morphine. J. Neurosci..

[66] Bodhankar S., Lapato A., Chen Y., Vandenbark A.A., Saugstad J.A., Offner H. (2015). Role for microglia in sex differences after ischemic stroke: Importance of M2. Metab. Brain Dis..

[67] Smith A.L., Alexander M., Rosenkrantz T.S., Sadek M.L., Fitch R.H. (2014). Sex differences in behavioral outcome following neonatal hypoxia ischemia: Insights from a clinical meta-analysis and a rodent model of induced hypoxic ischemic brain injury. Exp. Neurol..

[68] Demarest T.G., Schuh R.A., Waddell J., McKenna M.C., Fiskum G. (2016). Sex-dependent mitochondrial respiratory impairment and oxidative stress in a rat model of neonatal hypoxic-ischemic encephalopathy. J. Neurochem..

[69] Mrdjen D., Pavlovic A., Hartmann F.J., Schreiner B., Utz S.G., Leung B.P., Lelios I., Heppner F.L., Kipnis J., Merkler D. (2018). High-Dimensional Single-Cell Mapping of Central Nervous System Immune Cells Reveals Distinct Myeloid Subsets in Health, Aging, and Disease. Immunity.

[70] Manwani B., Liu F., Scranton V., Hammond M.D., Sansing L.H., McCullough L.D. (2013). Differential effects of aging and sex on stroke induced inflammation across the lifespan. Exp. Neurol..

[71] Acaz-Fonseca E., Duran J.C., Carrero P., Garcia-Segura L.M., Arevalo M.A. (2015). Sex differences in glia reactivity after cortical brain injury. Glia.

[72] Murphy S.J., McCullough L.D., Smith J.M. (2004). Stroke in the female: Role of biological sex and estrogen. ILAR J..

[73] Altmann A., Tian L., Henderson V.W., Greicius M.D. (2014). Alzheimer’s Disease Neuroimaging Initiative, I. Sex modifies the APOE-related risk of developing Alzheimer disease. Ann. Neurol..

[74] Hohman T.J., Dumitrescu L., Barnes L.L., Thambisetty M., Beecham G., Kunkle B., Gifford K.A., Bush W.S., Chibnik L.B., Mukherjee S. (2018). Sex-Specific Association of Apolipoprotein E With Cerebrospinal Fluid Levels of Tau. JAMA Neurol..

[75] Farrer L.A., Cupples L.A., Haines J.L., Hyman B., Kukull W.A., Mayeux R., Myers R.H., Pericak-Vance M.A., Risch N., van Duijn C.M. (1997). Effects of age, sex, and ethnicity on the association between apolipoprotein E genotype and Alzheimer disease. A meta-analysis. APOE and Alzheimer Disease Meta Analysis Consortium. JAMA.

[76] Bollinger J.L., Bergeon Burns C.M., Wellman C.L. (2016). Differential effects of stress on microglial cell activation in male and female medial prefrontal cortex. Brain Behav. Immun..

[77] Bollinger J.L., Collins K.E., Patel R., Wellman C.L. (2017). Behavioral stress alters corticolimbic microglia in a sex- and brain region-specific manner. PLoS ONE.

[78] Beggs S., Trang T., Salter M.W. (2012). P2X4R+ microglia drive neuropathic pain. Nat. Neurosci..

[79] Mapplebeck J.C., Beggs S., Salter M.W. (2017). Molecules in pain and sex: A developing story. Mol. Brain.

[80] Sorge R.E., Mapplebeck J.C., Rosen S., Beggs S., Taves S., Alexander J.K., Martin L.J., Austin J.S., Sotocinal S.G., Chen D. (2015). Different immune cells mediate mechanical pain hypersensitivity in male and female mice. Nat. Neurosci..

[81] Taves S., Berta T., Liu D.L., Gan S., Chen G., Kim Y.H., Van de Ven T., Laufer S., Ji R.R. (2016). Spinal inhibition of p38 MAP kinase reduces inflammatory and neuropathic pain in male but not female mice: Sex-dependent microglial signaling in the spinal cord. Brain Behav. Immun..

[82] Arias C., Zepeda A., Hernandez-Ortega K., Leal-Galicia P., Lojero C., Camacho-Arroyo I. (2009). Sex and estrous cycle-dependent differences in glial fibrillary acidic protein immunoreactivity in the adult rat hippocampus. Horm. Behav..

[83] Chowen J.A., Busiguina S., Garcia-Segura L.M. (1995). Sexual dimorphism and sex steroid modulation of glial fibrillary acidic protein messenger RNA and immunoreactivity levels in the rat hypothalamus. Neuroscience.

[84] Johnson R.T., Breedlove S.M., Jordan C.L. (2008). Sex differences and laterality in astrocyte number and complexity in the adult rat medial amygdala. J. Comp. Neurol..

[85] Mohr M.A., Garcia F.L., DonCarlos L.L., Sisk C.L. (2016). Neurons and Glial Cells Are Added to the Female Rat Anteroventral Periventricular Nucleus During Puberty. Endocrinology.

[86] Mong J.A., McCarthy M.M. (2002). Ontogeny of sexually dimorphic astrocytes in the neonatal rat arcuate. Brain Res. Dev. Brain Res..

[87] Mong J.A., Glaser E., McCarthy M.M. (1999). Gonadal steroids promote glial differentiation and alter neuronal morphology in the developing hypothalamus in a regionally specific manner. J. Neurosci..

[88] Acaz-Fonseca E., Ortiz-Rodriguez A., Azcoitia I., Garcia-Segura L.M., Arevalo M.A. (2019). Notch signaling in astrocytes mediates their morphological response to an inflammatory challenge. Cell Death Discov..

[89] Jaber S.M., Bordt E.A., Bhatt N.M., Lewis D.M., Gerecht S., Fiskum G., Polster B.M. (2018). Sex differences in the mitochondrial bioenergetics of astrocytes but not microglia at a physiologically relevant brain oxygen tension. Neurochem. Int..

[90] Crespo-Castrillo A., Garcia-Segura L.M., Arevalo M.A. (2020). The synthetic steroid tibolone exerts sex-specific regulation of astrocyte phagocytosis under basal conditions and after an inflammatory challenge. J. Neuroinflammation.

[91] Hsu C., Hsieh Y.L., Ho M.L., Hsu H.K., Yu J.Y. (2001). Sexually dimorphic effect of glutamate treatment on cell cycle arrestment of astrocytes from the preoptic area of neonatal rats. Dev. Neurosci..

[92] Morizawa Y., Sato K., Takaki J., Kawasaki A., Shibata K., Suzuki T., Ohta S., Koizumi S. (2012). Cell-autonomous enhancement of glutamate-uptake by female astrocytes. Cell. Mol. Neurobiol..

[93] Morselli E., Fuente-Martin E., Finan B., Kim M., Frank A., Garcia-Caceres C., Navas C.R., Gordillo R., Neinast M., Kalainayakan S.P. (2014). Hypothalamic PGC-1alpha protects against high-fat diet exposure by regulating ERalpha. Cell Rep..

[94] Morrison H.W., Filosa J.A. (2016). Sex differences in astrocyte and microglia responses immediately following middle cerebral artery occlusion in adult mice. Neuroscience.

[95] Cordeau P., Lalancette-Hebert M., Weng Y.C., Kriz J. (2008). Live imaging of neuroinflammation reveals sex and estrogen effects on astrocyte response to ischemic injury. Stroke.

[96] Ahnstedt H., Patrizz A., Chauhan A., Roy-O’Reilly M., Furr J.W., Spychala M.S., D’Aigle J., Blixt F.W., Zhu L., Bravo Alegria J. (2020). Sex differences in T cell immune responses, gut permeability and outcome after ischemic stroke in aged mice. Brain Behav. Immun..

[97] Santos-Galindo M., Acaz-Fonseca E., Bellini M.J., Garcia-Segura L.M. (2011). Sex differences in the inflammatory response of primary astrocytes to lipopolysaccharide. Biol. Sex. Differ..

[98] McCarthy M.M., Wright C.L., Schwarz J.M. (2009). New tricks by an old dogma: Mechanisms of the Organizational/Activational Hypothesis of steroid-mediated sexual differentiation of brain and behavior. Horm. Behav..

[99] Fernandez-Arjona M.D.M., Grondona J.M., Granados-Duran P., Fernandez-Llebrez P., Lopez-Avalos M.D. (2017). Microglia Morphological Categorization in a Rat Model of Neuroinflammation by Hierarchical Cluster and Principal Components Analysis. Front. Cell. Neurosci..

[100] Dubbelaar M.L., Kracht L., Eggen B.J.L., Boddeke E. (2018). The Kaleidoscope of Microglial Phenotypes. Front. Immunol..

[101] Petersen M.A., Dailey M.E. (2004). Diverse microglial motility behaviors during clearance of dead cells in hippocampal slices. Glia.

[102] Streit W.J., Walter S.A., Pennell N.A. (1999). Reactive microgliosis. Prog. Neurobiol..

[103] Sierra A., de Castro F., Del Rio-Hortega J., Rafael Iglesias-Rozas J., Garrosa M., Kettenmann H. (2016). The “Big-Bang” for modern glial biology: Translation and comments on Pio del Rio-Hortega 1919 series of papers on microglia. Glia.

[104] Kettenmann H., Hanisch U.K., Noda M., Verkhratsky A. (2011). Physiology of microglia. Physiol. Rev..

[105] Davalos D., Grutzendler J., Yang G., Kim J.V., Zuo Y., Jung S., Littman D.R., Dustin M.L., Gan W.B. (2005). ATP mediates rapid microglial response to local brain injury in vivo. Nat. Neurosci..

[106] Lee S.W., Gajavelli S., Spurlock M.S., Andreoni C., de Rivero Vaccari J.P., Bullock M.R., Keane R.W., Dietrich W.D. (2018). Microglial Inflammasome Activation in Penetrating Ballistic-Like Brain Injury. J. Neurotrauma.

[107] Turtzo L.C., Lescher J., Janes L., Dean D.D., Budde M.D., Frank J.A. (2014). Macrophagic and microglial responses after focal traumatic brain injury in the female rat. J. Neuroinflammation.

[108] Abdanipour A., Tiraihi T., Taheri T., Kazemi H. (2013). Microglial activation in rat experimental spinal cord injury model. Iran. Biomed. J..

[109] Thawer S.G., Mawhinney L., Chadwick K., de Chickera S.N., Weaver L.C., Brown A., Dekaban G.A. (2013). Temporal changes in monocyte and macrophage subsets and microglial macrophages following spinal cord injury in the Lys-Egfp-ki mouse model. J. Neuroimmunol..

[110] Carbonell W.S., Murase S., Horwitz A.F., Mandell J.W. (2005). Migration of perilesional microglia after focal brain injury and modulation by CC chemokine receptor 5: An in situ time-lapse confocal imaging study. J. Neurosci..

[111] De Simone R., Niturad C.E., De Nuccio C., Ajmone-Cat M.A., Visentin S., Minghetti L. (2010). TGF-beta and LPS modulate ADP-induced migration of microglial cells through P2Y1 and P2Y12 receptor expression. J. Neurochem..

[112] Ohsawa K., Irino Y., Nakamura Y., Akazawa C., Inoue K., Kohsaka S. (2007). Involvement of P2X4 and P2Y12 receptors in ATP-induced microglial chemotaxis. Glia.

[113] Cserep C., Posfai B., Lenart N., Fekete R., Laszlo Z.I., Lele Z., Orsolits B., Molnar G., Heindl S., Schwarcz A.D. (2020). Microglia monitor and protect neuronal function through specialized somatic purinergic junctions. Science.

[114] El Khoury J., Toft M., Hickman S.E., Means T.K., Terada K., Geula C., Luster A.D. (2007). Ccr2 deficiency impairs microglial accumulation and accelerates progression of Alzheimer-like disease. Nat. Med..

[115] Bianchi R., Kastrisianaki E., Giambanco I., Donato R. (2011). S100B protein stimulates microglia migration via RAGE-dependent up-regulation of chemokine expression and release. J. Biol. Chem..

[116] Louboutin J.P., Strayer D.S. (2013). Relationship between the chemokine receptor CCR5 and microglia in neurological disorders: Consequences of targeting CCR5 on neuroinflammation, neuronal death and regeneration in a model of epilepsy. CNS Neurol. Disord. Drug Targets.

[117] Koizumi S., Shigemoto-Mogami Y., Nasu-Tada K., Shinozaki Y., Ohsawa K., Tsuda M., Joshi B.V., Jacobson K.A., Kohsaka S., Inoue K. (2007). UDP acting at P2Y6 receptors is a mediator of microglial phagocytosis. Nature.

[118] Xu Y., Hu W., Liu Y., Xu P., Li Z., Wu R., Shi X., Tang Y. (2016). P2Y6 Receptor-Mediated Microglial Phagocytosis in Radiation-Induced Brain Injury. Mol. Neurobiol..

[119] Ruitenberg M.J., Vukovic J., Blomster L., Hall J.M., Jung S., Filgueira L., McMenamin P.G., Plant G.W. (2008). CX3CL1/fractalkine regulates branching and migration of monocyte-derived cells in the mouse olfactory epithelium. J. Neuroimmunol..

[120] Liang K.J., Lee J.E., Wang Y.D., Ma W., Fontainhas A.M., Fariss R.N., Wong W.T. (2009). Regulation of dynamic behavior of retinal microglia by CX3CR1 signaling. Invest. Ophthalmol. Vis. Sci..

[121] Cardona A.E., Pioro E.P., Sasse M.E., Kostenko V., Cardona S.M., Dijkstra I.M., Huang D., Kidd G., Dombrowski S., Dutta R. (2006). Control of microglial neurotoxicity by the fractalkine receptor. Nat. Neurosci..

[122] Harrison J.K., Jiang Y., Chen S., Xia Y., Maciejewski D., McNamara R.K., Streit W.J., Salafranca M.N., Adhikari S., Thompson D.A. (1998). Role for neuronally derived fractalkine in mediating interactions between neurons and CX3CR1-expressing microglia. Proc. Natl. Acad. Sci. USA.

[123] Jung S., Aliberti J., Graemmel P., Sunshine M.J., Kreutzberg G.W., Sher A., Littman D.R. (2000). Analysis of fractalkine receptor CX(3)CR1 function by targeted deletion and green fluorescent protein reporter gene insertion. Mol. Cell. Biol..

[124] Hatori K., Nagai A., Heisel R., Ryu J.K., Kim S.U. (2002). Fractalkine and fractalkine receptors in human neurons and glial cells. J. Neurosci. Res..

[125] Sokolowski J.D., Chabanon-Hicks C.N., Han C.Z., Heffron D.S., Mandell J.W. (2014). Fractalkine is a “find-me” signal released by neurons undergoing ethanol-induced apoptosis. Front. Cell. Neurosci..

[126] Lindia J.A., McGowan E., Jochnowitz N., Abbadie C. (2005). Induction of CX3CL1 expression in astrocytes and CX3CR1 in microglia in the spinal cord of a rat model of neuropathic pain. J. Pain.

[127] Zhu W., Acosta C., MacNeil B., Cortes C., Intrater H., Gong Y., Namaka M. (2013). Elevated expression of fractalkine (CX3CL1) and fractalkine receptor (CX3CR1) in the dorsal root ganglia and spinal cord in experimental autoimmune encephalomyelitis: Implications in multiple sclerosis-induced neuropathic pain. Biomed. Res. Int..

[128] Hsieh C.L., Koike M., Spusta S.C., Niemi E.C., Yenari M., Nakamura M.C., Seaman W.E. (2009). A role for TREM2 ligands in the phagocytosis of apoptotic neuronal cells by microglia. J. Neurochem..

[129] Takahashi K., Rochford C.D., Neumann H. (2005). Clearance of apoptotic neurons without inflammation by microglial triggering receptor expressed on myeloid cells-2. J. Exp. Med..

[130] Kleinberger G., Yamanishi Y., Suarez-Calvet M., Czirr E., Lohmann E., Cuyvers E., Struyfs H., Pettkus N., Wenninger-Weinzierl A., Mazaheri F. (2014). TREM2 mutations implicated in neurodegeneration impair cell surface transport and phagocytosis. Sci. Transl. Med..

[131] Yeh F.L., Hansen D.V., Sheng M. (2017). TREM2, Microglia, and Neurodegenerative Diseases. Trends Mol. Med..

[132] Suarez-Calvet M., Kleinberger G., Araque Caballero M.A., Brendel M., Rominger A., Alcolea D., Fortea J., Lleo A., Blesa R., Gispert J.D. (2016). sTREM2 cerebrospinal fluid levels are a potential biomarker for microglia activity in early-stage Alzheimer’s disease and associate with neuronal injury markers. EMBO Mol. Med..

[133] Jones R.S., Minogue A.M., Connor T.J., Lynch M.A. (2013). Amyloid-beta-induced astrocytic phagocytosis is mediated by CD36, CD47 and RAGE. J. Neuroimmune Pharmacol..

[134] Chung W.S., Allen N.J., Eroglu C. (2015). Astrocytes Control Synapse Formation, Function, and Elimination. Cold Spring Harb. Perspect. Biol..

[135] Sokolowski J.D., Mandell J.W. (2011). Phagocytic clearance in neurodegeneration. Am. J. Pathol..

[136] Rahimian R., Cordeau P., Kriz J. (2019). Brain Response to Injuries: When Microglia Go Sexist. Neuroscience.

[137] Bass N.H., Hess H.H., Pope A., Thalheimer C. (1971). Quantitative cytoarchitectonic distribution of neurons, glia, and DNa in rat cerebral cortex. J. Comp. Neurol..

[138] Gengatharan A., Bammann R.R., Saghatelyan A. (2016). The Role of Astrocytes in the Generation, Migration, and Integration of New Neurons in the Adult Olfactory Bulb. Front. Neurosci..

[139] Alvarez-Buylla A., Garcia-Verdugo J.M., Tramontin A.D. (2001). A unified hypothesis on the lineage of neural stem cells. Nat. Rev. Neurosci..

[140] Araque A., Parpura V., Sanzgiri R.P., Haydon P.G. (1999). Tripartite synapses: Glia, the unacknowledged partner. Trends Neurosci..

[141] Sun W., McConnell E., Pare J.F., Xu Q., Chen M., Peng W., Lovatt D., Han X., Smith Y., Nedergaard M. (2013). Glutamate-dependent neuroglial calcium signaling differs between young and adult brain. Science.

[142] Farhy-Tselnicker I., Allen N.J. (2018). Astrocytes, neurons, synapses: A tripartite view on cortical circuit development. Neural Dev..

[143] Chung W.S., Clarke L.E., Wang G.X., Stafford B.K., Sher A., Chakraborty C., Joung J., Foo L.C., Thompson A., Chen C. (2013). Astrocytes mediate synapse elimination through MEGF10 and MERTK pathways. Nature.

[144] Jung Y.J., Chung W.S. (2018). Phagocytic Roles of Glial Cells in Healthy and Diseased Brains. Biomol. Ther. (Seoul).

[145] Park D., Tosello-Trampont A.C., Elliott M.R., Lu M., Haney L.B., Ma Z., Klibanov A.L., Mandell J.W., Ravichandran K.S. (2007). BAI1 is an engulfment receptor for apoptotic cells upstream of the ELMO/Dock180/Rac module. Nature.

[146] Elliott M.R., Ravichandran K.S. (2010). Clearance of apoptotic cells: Implications in health and disease. J. Cell Biol..

[147] Weber B., Barros L.F. (2015). The Astrocyte: Powerhouse and Recycling Center. Cold Spring Harb. Perspect. Biol..

[148] Almutairi M.M., Gong C., Xu Y.G., Chang Y., Shi H. (2016). Factors controlling permeability of the blood-brain barrier. Cell Mol. Life Sci..

[149] McCarthy M.M., Todd B.J., Amateau S.K. (2003). Estradiol modulation of astrocytes and the establishment of sex differences in the brain. Ann. N. Y. Acad. Sci..

[150] Kuo J., Hamid N., Bondar G., Dewing P., Clarkson J., Micevych P. (2010). Sex differences in hypothalamic astrocyte response to estradiol stimulation. Biol. Sex Differ..

[151] Sofroniew M.V. (2014). Astrogliosis. Cold Spring Harb. Perspect. Biol..

[152] Sofroniew M.V., Vinters H.V. (2010). Astrocytes: Biology and pathology. Acta Neuropathol..

[153] Vainchtein I.D., Molofsky A.V. (2020). Astrocytes and Microglia: In Sickness and in Health. Trends Neurosci..

[154] Liddelow S.A., Guttenplan K.A., Clarke L.E., Bennett F.C., Bohlen C.J., Schirmer L., Bennett M.L., Munch A.E., Chung W.S., Peterson T.C. (2017). Neurotoxic reactive astrocytes are induced by activated microglia. Nature.

[155] Sofroniew M.V. (2009). Molecular dissection of reactive astrogliosis and glial scar formation. Trends Neurosci..

[156] Mierzwa A.J., Marion C.M., Sullivan G.M., McDaniel D.P., Armstrong R.C. (2015). Components of myelin damage and repair in the progression of white matter pathology after mild traumatic brain injury. J. Neuropathol. Exp. Neurol..

[157] Dutta R., Trapp B.D. (2011). Mechanisms of neuronal dysfunction and degeneration in multiple sclerosis. Prog. Neurobiol..

[158] Orr M.B., Gensel J.C. (2018). Spinal Cord Injury Scarring and Inflammation: Therapies Targeting Glial and Inflammatory Responses. Neurotherapeutics.

[159] Gaudet A.D., Fonken L.K. (2018). Glial Cells Shape Pathology and Repair After Spinal Cord Injury. Neurotherapeutics.

[160] Burda J.E., Sofroniew M.V. (2014). Reactive gliosis and the multicellular response to CNS damage and disease. Neuron.

[161] Adams K.L., Gallo V. (2018). The diversity and disparity of the glial scar. Nat. Neurosci..

[162] Cekanaviciute E., Fathali N., Doyle K.P., Williams A.M., Han J., Buckwalter M.S. (2014). Astrocytic transforming growth factor-beta signaling reduces subacute neuroinflammation after stroke in mice. Glia.

[163] Li S., Gu X., Yi S. (2017). The Regulatory Effects of Transforming Growth Factor-beta on Nerve Regeneration. Cell Transplant..

[164] Schachtrup C., Ryu J.K., Helmrick M.J., Vagena E., Galanakis D.K., Degen J.L., Margolis R.U., Akassoglou K. (2010). Fibrinogen triggers astrocyte scar formation by promoting the availability of active TGF-beta after vascular damage. J. Neurosci..

[165] Diniz L.P., Tortelli V., Matias I., Morgado J., Bergamo Araujo A.P., Melo H.M., Seixas da Silva G.S., Alves-Leon S.V., de Souza J.M., Ferreira S.T. (2017). Astrocyte Transforming Growth Factor Beta 1 Protects Synapses against Abeta Oligomers in Alzheimer’s Disease Model. J. Neurosci..

[166] Herrmann J.E., Imura T., Song B., Qi J., Ao Y., Nguyen T.K., Korsak R.A., Takeda K., Akira S., Sofroniew M.V. (2008). STAT3 is a critical regulator of astrogliosis and scar formation after spinal cord injury. J. Neurosci..

[167] Poyhonen S., Er S., Domanskyi A., Airavaara M. (2019). Effects of Neurotrophic Factors in Glial Cells in the Central Nervous System: Expression and Properties in Neurodegeneration and Injury. Front. Physiol..

[168] Faulkner J.R., Herrmann J.E., Woo M.J., Tansey K.E., Doan N.B., Sofroniew M.V. (2004). Reactive astrocytes protect tissue and preserve function after spinal cord injury. J. Neurosci..

[169] Neumann H., Kotter M.R., Franklin R.J. (2009). Debris clearance by microglia: An essential link between degeneration and regeneration. Brain.

[170] Dewing P., Shi T., Horvath S., Vilain E. (2003). Sexually dimorphic gene expression in mouse brain precedes gonadal differentiation. Brain Res. Mol. Brain Res..

[171] Cui J., Shen Y., Li R. (2013). Estrogen synthesis and signaling pathways during aging: From periphery to brain. Trends Mol. Med..

[172] Soldan S.S., Alvarez Retuerto A.I., Sicotte N.L., Voskuhl R.R. (2003). Immune modulation in multiple sclerosis patients treated with the pregnancy hormone estriol. J. Immunol..

[173] Gatson J.W., Liu M.M., Abdelfattah K., Wigginton J.G., Smith S., Wolf S., Simpkins J.W., Minei J.P. (2012). Estrone is neuroprotective in rats after traumatic brain injury. J. Neurotrauma.

[174] Garcia-Segura L.M., Azcoitia I., DonCarlos L.L. (2001). Neuroprotection by estradiol. Prog. Neurobiol..

[175] Lejri I., Grimm A., Eckert A. (2018). Mitochondria, Estrogen and Female Brain Aging. Front. Aging Neurosci..

[176] Gillies G.E., McArthur S. (2010). Estrogen actions in the brain and the basis for differential action in men and women: A case for sex-specific medicines. Pharmacol. Rev..

[177] McCarthy M.M., Arnold A.P., Ball G.F., Blaustein J.D., De Vries G.J. (2012). Sex differences in the brain: The not so inconvenient truth. J. Neurosci..

[178] Sinchak K., Wagner E.J. (2012). Estradiol signaling in the regulation of reproduction and energy balance. Front. Neuroendocrinol..

[179] Barakat R., Oakley O., Kim H., Jin J., Ko C.J. (2016). Extra-gonadal sites of estrogen biosynthesis and function. BMB Rep..

[180] Do Rego J.L., Seong J.Y., Burel D., Leprince J., Luu-The V., Tsutsui K., Tonon M.C., Pelletier G., Vaudry H. (2009). Neurosteroid biosynthesis: Enzymatic pathways and neuroendocrine regulation by neurotransmitters and neuropeptides. Front. Neuroendocrinol..

[181] Rosenfeld C.S., Shay D.A., Vieira-Potter V.J. (2018). Cognitive Effects of Aromatase and Possible Role in Memory Disorders. Front. Endocrinol. (Lausanne).

[182] Garcia-Segura L.M., Wozniak A., Azcoitia I., Rodriguez J.R., Hutchison R.E., Hutchison J.B. (1999). Aromatase expression by astrocytes after brain injury: Implications for local estrogen formation in brain repair. Neuroscience.

[183] Shang Y., Hu X., DiRenzo J., Lazar M.A., Brown M. (2000). Cofactor dynamics and sufficiency in estrogen receptor-regulated transcription. Cell.

[184] Kelly M.J., Levin E.R. (2001). Rapid actions of plasma membrane estrogen receptors. Trends Endocrinol. Metab..

[185] Crider A., Pillai A. (2017). Estrogen Signaling as a Therapeutic Target in Neurodevelopmental Disorders. J. Pharmacol. Exp. Ther..

[186] Jia M., Dahlman-Wright K., Gustafsson J.A. (2015). Estrogen receptor alpha and beta in health and disease. Best Pract. Res. Clin. Endocrinol. Metab..

[187] Barros R.P., Gustafsson J.A. (2011). Estrogen receptors and the metabolic network. Cell Metab..

[188] Saczko J., Michel O., Chwilkowska A., Sawicka E., Maczynska J., Kulbacka J. (2017). Estrogen Receptors in Cell Membranes: Regulation and Signaling. Adv. Anat. Embryol. Cell Biol..

[189] Hutson D.D., Gurrala R., Ogola B.O., Zimmerman M.A., Mostany R., Satou R., Lindsey S.H. (2019). Estrogen receptor profiles across tissues from male and female Rattus norvegicus. Biol. Sex Differ..

[190] Reddy R.C., Estill C.T., Meaker M., Stormshak F., Roselli C.E. (2014). Sex differences in expression of oestrogen receptor alpha but not androgen receptor mRNAs in the foetal lamb brain. J. Neuroendocrinol..

[191] Yasar P., Ayaz G., User S.D., Gupur G., Muyan M. (2017). Molecular mechanism of estrogen-estrogen receptor signaling. Reprod. Med. Biol..

[192] Le Romancer M., Poulard C., Cohen P., Sentis S., Renoir J.M., Corbo L. (2011). Cracking the estrogen receptor’s posttranslational code in breast tumors. Endocr. Rev..

[193] Garcia-Ovejero D., Veiga S., Garcia-Segura L.M., Doncarlos L.L. (2002). Glial expression of estrogen and androgen receptors after rat brain injury. J. Comp. Neurol..

[194] Wilson M.E., Rosewell K.L., Kashon M.L., Shughrue P.J., Merchenthaler I., Wise P.M. (2002). Age differentially influences estrogen receptor-alpha (ERalpha) and estrogen receptor-beta (ERbeta) gene expression in specific regions of the rat brain. Mech. Ageing Dev..

[195] Roque C., Mendes-Oliveira J., Baltazar G. (2019). G protein-coupled estrogen receptor activates cell type-specific signaling pathways in cortical cultures: Relevance to the selective loss of astrocytes. J. Neurochem..

[196] Vegeto E., Bonincontro C., Pollio G., Sala A., Viappiani S., Nardi F., Brusadelli A., Viviani B., Ciana P., Maggi A. (2001). Estrogen prevents the lipopolysaccharide-induced inflammatory response in microglia. J. Neurosci..

[197] Pawlak J., Brito V., Kuppers E., Beyer C. (2005). Regulation of glutamate transporter GLAST and GLT-1 expression in astrocytes by estrogen. Brain Res. Mol. Brain Res..

[198] Liu T., Zhang L., Joo D., Sun S.C. (2017). NF-kappaB signaling in inflammation. Signal Transduct. Target. Ther..

[199] Karki P., Smith K., Johnson J., Lee E. (2014). Astrocyte-derived growth factors and estrogen neuroprotection: Role of transforming growth factor-alpha in estrogen-induced upregulation of glutamate transporters in astrocytes. Mol. Cell. Endocrinol..

[200] Spence R.D., Wisdom A.J., Cao Y., Hill H.M., Mongerson C.R., Stapornkul B., Itoh N., Sofroniew M.V., Voskuhl R.R. (2013). Estrogen mediates neuroprotection and anti-inflammatory effects during EAE through ERalpha signaling on astrocytes but not through ERbeta signaling on astrocytes or neurons. J. Neurosci..

[201] Giraud S.N., Caron C.M., Pham-Dinh D., Kitabgi P., Nicot A.B. (2010). Estradiol inhibits ongoing autoimmune neuroinflammation and NFkappaB-dependent CCL2 expression in reactive astrocytes. Proc. Natl. Acad. Sci. USA.

[202] De Marinis E., Fiocchetti M., Acconcia F., Ascenzi P., Marino M. (2013). Neuroglobin upregulation induced by 17beta-estradiol sequesters cytocrome c in the mitochondria preventing H2O2-induced apoptosis of neuroblastoma cells. Cell Death Dis..

[203] Duenas M., Luquin S., Chowen J.A., Torres-Aleman I., Naftolin F., Garcia-Segura L.M. (1994). Gonadal hormone regulation of insulin-like growth factor-I-like immunoreactivity in hypothalamic astroglia of developing and adult rats. Neuroendocrinology.

[204] Lee E., Sidoryk-Wegrzynowicz M., Wang N., Webb A., Son D.S., Lee K., Aschner M. (2012). GPR30 regulates glutamate transporter GLT-1 expression in rat primary astrocytes. J. Biol. Chem..

[205] Frago L.M., Canelles S., Freire-Regatillo A., Argente-Arizon P., Barrios V., Argente J., Garcia-Segura L.M., Chowen J.A. (2017). Estradiol Uses Different Mechanisms in Astrocytes from the Hippocampus of Male and Female Rats to Protect against Damage Induced by Palmitic Acid. Front. Mol. Neurosci..

[206] Bruce-Keller A.J., Keeling J.L., Keller J.N., Huang F.F., Camondola S., Mattson M.P. (2000). Antiinflammatory effects of estrogen on microglial activation. Endocrinology.

[207] Sierra A., Gottfried-Blackmore A., Milner T.A., McEwen B.S., Bulloch K. (2008). Steroid hormone receptor expression and function in microglia. Glia.

[208] Wu W.F., Tan X.J., Dai Y.B., Krishnan V., Warner M., Gustafsson J.A. (2013). Targeting estrogen receptor beta in microglia and T cells to treat experimental autoimmune encephalomyelitis. Proc. Natl. Acad. Sci. USA.

[209] Ghisletti S., Meda C., Maggi A., Vegeto E. (2005). 17beta-estradiol inhibits inflammatory gene expression by controlling NF-kappaB intracellular localization. Mol. Cell. Biol..

[210] Murphy A.J., Guyre P.M., Pioli P.A. (2010). Estradiol suppresses NF-kappa B activation through coordinated regulation of let-7a and miR-125b in primary human macrophages. J. Immunol..

[211] Chan P.H. (2001). Reactive oxygen radicals in signaling and damage in the ischemic brain. J. Cereb. Blood Flow Metab..

[212] Baker A.E., Brautigam V.M., Watters J.J. (2004). Estrogen modulates microglial inflammatory mediator production via interactions with estrogen receptor beta. Endocrinology.

[213] Zhao T.Z., Ding Q., Hu J., He S.M., Shi F., Ma L.T. (2016). GPER expressed on microglia mediates the anti-inflammatory effect of estradiol in ischemic stroke. Brain Behav..

[214] Perez-Pouchoulen M., Yu S.J., Roby C.R., Bonsavage N., McCarthy M.M. (2019). Regulatory Control of Microglial Phagocytosis by Estradiol and Prostaglandin E2 in the Developing Rat Cerebellum. Cerebellum.

[215] Vegeto E., Benedusi V., Maggi A. (2008). Estrogen anti-inflammatory activity in brain: A therapeutic opportunity for menopause and neurodegenerative diseases. Front. Neuroendocrinol..

[216] Zarate S., Stevnsner T., Gredilla R. (2017). Role of Estrogen and Other Sex Hormones in Brain Aging. Neuroprotection and DNA Repair. Front. Aging Neurosci..

[217] Arevalo M.A., Diz-Chaves Y., Santos-Galindo M., Bellini M.J., Garcia-Segura L.M. (2012). Selective oestrogen receptor modulators decrease the inflammatory response of glial cells. J. Neuroendocrinol..

[218] Garcia-Sanchis L., Lopez-Aznar D., Oltra A., Rivas A., Alonso J., Montalar J., Mateo A. (1999). Metastatic follicular thyroid carcinoma to the kidney: A case report. Clin. Nucl. Med..

[219] Jurado-Coronel J.C., Cabezas R., Avila Rodriguez M.F., Echeverria V., Garcia-Segura L.M., Barreto G.E. (2018). Sex differences in Parkinson’s disease: Features on clinical symptoms, treatment outcome, sexual hormones and genetics. Front. Neuroendocrinol..

[220] Arevalo M.A., Azcoitia I., Garcia-Segura L.M. (2015). The neuroprotective actions of oestradiol and oestrogen receptors. Nat. Rev. Neurosci..

[221] Selvamani A., Sohrabji F. (2010). Reproductive age modulates the impact of focal ischemia on the forebrain as well as the effects of estrogen treatment in female rats. Neurobiol. Aging.

[222] Leon R.L., Li X., Huber J.D., Rosen C.L. (2012). Worsened outcome from middle cerebral artery occlusion in aged rats receiving 17beta-estradiol. Endocrinology.

[223] De Butte-Smith M., Nguyen A.P., Zukin R.S., Etgen A.M., Colbourne F. (2007). Failure of estradiol to ameliorate global ischemia-induced CA1 sector injury in middle-aged female gerbils. Brain Res..

[224] Chuffa L.G., Lupi-Junior L.A., Costa A.B., Amorim J.P., Seiva F.R. (2017). The role of sex hormones and steroid receptors on female reproductive cancers. Steroids.

[225] McKenna N.J., O’Malley B.W. (2000). An issue of tissues: Divining the split personalities of selective estrogen receptor modulators. Nat. Med..

[226] Tapia-Gonzalez S., Carrero P., Pernia O., Garcia-Segura L.M., Diz-Chaves Y. (2008). Selective oestrogen receptor (ER) modulators reduce microglia reactivity in vivo after peripheral inflammation: Potential role of microglial ERs. J. Endocrinol..

[227] Cerciat M., Unkila M., Garcia-Segura L.M., Arevalo M.A. (2010). Selective estrogen receptor modulators decrease the production of interleukin-6 and interferon-gamma-inducible protein-10 by astrocytes exposed to inflammatory challenge in vitro. Glia.

[228] Barreto G.E., Santos-Galindo M., Garcia-Segura L.M. (2014). Selective estrogen receptor modulators regulate reactive microglia after penetrating brain injury. Front. Aging Neurosci..

[229] Li R., Xu W., Chen Y., Qiu W., Shu Y., Wu A., Dai Y., Bao J., Lu Z., Hu X. (2014). Raloxifene suppresses experimental autoimmune encephalomyelitis and NF-kappaB-dependent CCL20 expression in reactive astrocytes. PLoS ONE.

[230] Mosquera L., Colon J.M., Santiago J.M., Torrado A.I., Melendez M., Segarra A.C., Rodriguez-Orengo J.F., Miranda J.D. (2014). Tamoxifen and estradiol improved locomotor function and increased spared tissue in rats after spinal cord injury: Their antioxidant effect and role of estrogen receptor alpha. Brain Res..

[231] Ishihara Y., Itoh K., Ishida A., Yamazaki T. (2015). Selective estrogen-receptor modulators suppress microglial activation and neuronal cell death via an estrogen receptor-dependent pathway. J. Steroid Biochem. Mol. Biol..

[232] Gennari L., Merlotti D., Nuti R. (2010). Selective estrogen receptor modulator (SERM) for the treatment of osteoporosis in postmenopausal women: Focus on lasofoxifene. Clin. Interv. Aging.

[233] Jordan V.C. (2007). Chemoprevention of breast cancer with selective oestrogen-receptor modulators. Nat. Rev. Cancer.

[234] DonCarlos L.L., Azcoitia I., Garcia-Segura L.M. (2009). Neuroprotective actions of selective estrogen receptor modulators. Psychoneuroendocrinology.

[235] Lien E.A., Solheim E., Ueland P.M. (1991). Distribution of tamoxifen and its metabolites in rat and human tissues during steady-state treatment. Cancer Res..

[236] Pareto D., Alvarado M., Hanrahan S.M., Biegon A. (2004). In vivo occupancy of female rat brain estrogen receptors by 17beta-estradiol and tamoxifen. Neuroimage.

[237] Wu X., Glinn M.A., Ostrowski N.L., Su Y., Ni B., Cole H.W., Bryant H.U., Paul S.M. (1999). Raloxifene and estradiol benzoate both fully restore hippocampal choline acetyltransferase activity in ovariectomized rats. Brain Res..

[238] Reed M.J., Kloosterboer H.J. (2004). Tibolone: A selective tissue estrogenic activity regulator (STEAR). Maturitas.

[239] Kloosterboer H.J. (2001). Tibolone: A steroid with a tissue-specific mode of action. J. Steroid Biochem. Mol. Biol..

[240] Kloosterboer H.J. (2004). Tissue-selectivity: The mechanism of action of tibolone. Maturitas.

[241] Timmer C.J., Verheul H.A., Doorstam D.P. (2002). Pharmacokinetics of tibolone in early and late postmenopausal women. Br. J. Clin. Pharmacol..

[242] Verheul H.A., Kloosterboer H.J. (2006). Metabolism of exogenous sex steroids and effect on brain functions with a focus on tibolone. J. Steroid Biochem. Mol. Biol..

[243] Behl C., Skutella T., Lezoualc’h F., Post A., Widmann M., Newton C.J., Holsboer F. (1997). Neuroprotection against oxidative stress by estrogens: Structure-activity relationship. Mol. Pharmacol..

[244] Crespo-Castrillo A., Yanguas-Casas N., Arevalo M.A., Azcoitia I., Barreto G.E., Garcia-Segura L.M. (2018). The Synthetic Steroid Tibolone Decreases Reactive Gliosis and Neuronal Death in the Cerebral Cortex of Female Mice After a Stab Wound Injury. Mol. Neurobiol..

[245] Avila Rodriguez M., Garcia-Segura L.M., Cabezas R., Torrente D., Capani F., Gonzalez J., Barreto G.E. (2014). Tibolone protects T98G cells from glucose deprivation. J. Steroid Biochem. Mol. Biol..

[246] Avila-Rodriguez M., Garcia-Segura L.M., Hidalgo-Lanussa O., Baez E., Gonzalez J., Barreto G.E. (2016). Tibolone protects astrocytic cells from glucose deprivation through a mechanism involving estrogen receptor beta and the upregulation of neuroglobin expression. Mol. Cell. Endocrinol..

[247] Hidalgo-Lanussa O., Avila-Rodriguez M., Baez-Jurado E., Zamudio J., Echeverria V., Garcia-Segura L.M., Barreto G.E. (2018). Tibolone Reduces Oxidative Damage and Inflammation in Microglia Stimulated with Palmitic Acid through Mechanisms Involving Estrogen Receptor Beta. Mol. Neurobiol..

